# Supplementary material for: Role of export industries on ozone pollution and its precursors in China
Source: Nat Commun. 2020 Oct 30;11:5492. doi: 10.1038/s41467-020-19035-x (PMC7603491; doi:10.1038/s41467-020-19035-x)
Supplement: Supplementary file 1 — Supplementary Information [file 41467_2020_19035_MOESM1_ESM.pdf]

Supplementary Information

Ou *et al*., Role of export industries on ozone pollution and its precursors in China

Table of Content

Supplementary Note 1 Case setting 2

Supplementary Note 2 Model configurations and validation 3

Supplementary Table 1 List of countries 4

Supplementary Table 2 Details on model configuration 8

Supplementary Table 3 Statistics of model performances 9

Supplementary Figure 1 Gross Domestic Product of export by provinces in 2013 10

Supplementary Figure 2 Export concentration to MAX 8h O_3 in (a) January and (b) April, 2013 11

Supplementary Figure 3 Change of BTEX and MAX 8h O_3 by cleaner production practices only in export capacities 12

Supplementary Figure 4 Locations of ambient ozone monitoring sites in China 13

Supplementary References 14

Supplementary Note 1 Case setting

To study the impact of export industries and the pathways to mitigate its footprint, this study sets up a few cases in the validated modeling platform. A base case and the other 3 cases were constructed. The differences between different bases were emission inputs.

For the base case, air pollutant emissions of NOx, NMVOCs and CO for the year of 2013 were adopted, which represented the 'true' emissions (emissions in reality under the best knowledge) in 2013. Case 1 to 3 used reconstructed emissions to either study the impact of export or the effectiveness of the proposed manners.

Case 1 was set up to study the impact of export-driven emissions on BTEX and O_3 concentration. In Case 1, NOx, NMVOCs, and CO emissions relevant to export demands were excluded. By comparing the modelling results from base case and Case 1, the contribution of export-driven emissions on O_3 and BTEX concentration can be revealed.

Case 2 was set up to test the effectiveness of the cleaner production manners (in line with the proven and affordable technologies in EU) in the export capacities. By comparing the sectoral emission intensities in China and EU, we estimated that an 1,165 kt of NMVOCs can be reduced from the export-relevant industrial capacities. Given that NOx have been reduced aggressively and the persistent growth of NMVOCs from industries, we focused on the reduction of NMVOCs in this study and did not extend the discussion to the reductions of other precursors. Therefore, an 1,165 kt of NMVOCs were excluded from the emissions inputs of Case 2, while the inputs of NOx and CO remained the same as that of base case. Please note that the reductions of NMVOCs were done by sectors. In other words, sectoral specific reduction was made instead of an even reduction of all sectors. By comparing the results of Case 2 and base case, we can estimate the effectiveness of the cleaner production manners in the export capacities.

Case 3 was developed to understand the wider impact of cleaner production manners. Case 2 assumed that cleaner production manners were only applied to export production lines. However, it might not be true in reality. If a shoe-making factory is decided to upgrade its technology and management, for example, it is more plausibly done for the whole production line rather than only for the shoes for export. We estimated that the proposed manners in Case 2 would lead to a reduction of 4,437 kt of NMVOCs if they targeted the whole production capacities. Therefore, Case 3 was set up by reducing the NMVOCs from industries by 4,437 kt, while the inputs of NOx and CO remained the same as that of base case. Results of Case 3 were compared with base case to fathom the effectiveness of an industry-wide NMVOCs reduction effort.

Supplementary Note 2 Model configurations and validation

The study domain of this work is mainland China, with a spatial resolution of 27km × 27km. The air quality modeling platform coupled the Weather Research and Forecast (WRF) model [1], SparseMatrix Operator Kernel Emissions (SMOKE) model [2], and CMAQ model [3]. The Weather Research and Forecast (WRF) model v3.9 was used to provide meteorological data. The CMAQ v5.0.2 with the CB-05 gas-phase chemical mechanism was used to simulate the ambient O_3 mixing ratios under different precursor emissions scenario. The SMOKE provided model-ready emission data by allocating the annual emissions at province level into hourly interval and grid cell. Species allocations were also involved. Take Case 1 as an example, the annual bulk emission inventory for the base year of 2013 is first developed. Given that the input-output table is also in an annual basis, the bulk emission inventory is used as an input for the environmentally-extended input-output (EEIO) analysis to produce the consumption-based emission inventory. The consumption-based emission inventory reveals that how many emissions in each source category are associated with the demand of export. By excluding the emissions driven by export, a new bulk emission inventory is generated for Case 1, which is the case for us to study the impact of export. The new bulk emission inventory is processed with the temporal and spatial surrogates and emission processing systems to have the monthly, daily and even hourly emission inputs for the air quality modelling system and other analysis. The model-ready meteorological and emission data was then fed into air quality model. The model was spin-up for 3 days in each month to eliminate the impact of initial conditions. Detailed model configurations of CMAQ and WRF are shown in Table 2.

Ground-level O_3 measurements were used to validate the modeling platform. The locations of ambient ozone monitoring sites are shown in Supplementary Fig.4. In China, ambient O_3 mixing ratios were not regularly measured nation-wide until 2013. The records of ambient O_3 from China's national air quality monitoring network were adopted for the reference year 2013. Specifically, the performance of modeling platform in July and October 2013 were evaluated. These two months represented two typical O_3 seasons in China. Normalized mean bias (NMB), normalized mean error (NME), and correlation coefficient (R) were used as indicators of model performance. According to recommended benchmarks for photochemical model performance statistics, the NMB for the 1 hour average or maximum daily 8 hour average ozone should be no larger than 15%, and the R should be higher than 0.50 [4]. The model performances of this work (See Supplementary Table 3) were within the above suggested range. The NME of this study was similar to those of previous studies in China [5]–[7]. For example, the NME for the 1 hour average O_3 over the eastern China in July was around 58.8~62.7% [7]. The modeling system can reproduce the O_3 mixing ratio reliably. In case study, this study mainly refers to the maximum 8 hour average since it was reproduced well in the model and it is more relevant to the health impact.

Supplementary Table 1 List of countries

Number in GTAP	Code	Description	World region
1	AUS	Australia	Developed regions in Asia and Pacific
2	NZL	New Zealand	Developed regions in Asia and Pacific
3	XOC	Rest of Oceania	Developing regions in Asia and Pacific
4	CHN	China	China
5	HKG	Hong Kong, Special Administrative Region of China	Developed regions in Asia and Pacific
6	JPN	Japan	Developed regions in Asia and Pacific
7	KOR	Korea, Republic of	Developed regions in Asia and Pacific
8	MNG	Mongolia	Developing regions in Asia and Pacific
9	TWN	Taiwan	Developed regions in Asia and Pacific
10	XEA	Rest of East Asia	Developing regions in Asia and Pacific
11	BRN	Brunei Darussalam	Developing regions in Asia and Pacific
12	KHM	Cambodia	Developing regions in Asia and Pacific
13	IDN	Indonesia	Developing regions in Asia and Pacific
14	LAO	Lao PDR	Developing regions in Asia and Pacific
15	MYS	Malaysia	Developing regions in Asia and Pacific
16	PHL	Philippines	Developing regions in Asia and Pacific
17	SGP	Singapore	Developed regions in Asia and Pacific
18	THA	Thailand	Developing regions in Asia and Pacific
19	VNM	Viet Nam	Developing regions in Asia and Pacific
20	XSE	Rest of Southeast Asia	Developing regions in Asia and Pacific
21	BGD	Bangladesh	Developing regions in Asia and Pacific
22	IND	India	India
23	NPL	Nepal	Developing regions in Asia and Pacific
24	PAK	Pakistan	Developing regions in Asia and Pacific
25	LKA	Sri Lanka	Developing regions in Asia and Pacific
26	XSA	Rest of South Asia	Developing regions in Asia and Pacific
27	CAN	Canada	North America (USA, Canada)
28	USA	United States of America	North America (USA, Canada)
29	MEX	Mexico	Latin America and Caribbean
30	XNA	Rest of North America	Latin America and Caribbean
31	ARG	Argentina	Latin America and Caribbean
32	BOL	Bolivia	Latin America and Caribbean
33	BRA	Brazil	Latin America and Caribbean
34	CHL	Chile	Latin America and Caribbean
35	COL	Colombia	Latin America and Caribbean
36	ECU	Ecuador	Latin America and Caribbean
37	PRY	Paraguay	Latin America and Caribbean
38	PER	Peru	Latin America and Caribbean
39	URY	Uruguay	Latin America and Caribbean
40	VEN	Venezuela (Bolivarian Republic	Latin America and Caribbean

		of)	
41	XSM	Rest of South America	Latin America and Caribbean
42	CRI	Costa Rica	Latin America and Caribbean
43	GTM	Guatemala	Latin America and Caribbean
44	HND	Honduras	Latin America and Caribbean
45	NIC	Nicaragua	Latin America and Caribbean
46	PAN	Panama	Latin America and Caribbean
47	SLV	El Salvador	Latin America and Caribbean
48	XCA	Rest of Central America	Latin America and Caribbean
49	DOM	Dominican Republic P	Latin America and Caribbean
50	JAM	Jamaica	Latin America and Caribbean
51	PRI	Puerto Rico	Latin America and Caribbean
52	TTO	Trinidad and Tobago P	Latin America and Caribbean
53	XCB	Rest of Caribbean	Latin America and Caribbean
54	AUT	Austria	Western Europe
55	BEL	Belgium	Western Europe
56	CYP	Cyprus	Western Europe
57	CZE	Czech Republic	Economies in Transition (Eastern Europe and former Soviet Union)
58	DNK	Denmark	Western Europe
59	EST	Estonia	Economies in Transition (Eastern Europe and former Soviet Union)
60	FIN	Finland	Western Europe
61	FRA	France	Western Europe
62	DEU	Germany	Western Europe
63	GRC	Greece	Western Europe
64	HUN	Hungary	Economies in Transition (Eastern Europe and former Soviet Union)
65	IRL	Ireland	Western Europe
66	ITA	Italy	Western Europe
67	LVA	Latvia	Economies in Transition (Eastern Europe and former Soviet Union)
68	LTU	Lithuania	Economies in Transition (Eastern Europe and former Soviet Union)
69	LUX	Luxembourg	Western Europe
70	MLT	Malta	Western Europe
71	NLD	Netherlands	Western Europe
72	POL	Poland	Economies in Transition (Eastern Europe and former Soviet Union)
73	PRT	Portugal	Western Europe
74	SVK	Slovakia	Economies in Transition (Eastern Europe and former Soviet Union)
75	SVN	Slovenia	Economies in Transition (Eastern Europe and former Soviet Union)
76	ESP	Spain	Western Europe
77	SWE	Sweden	Western Europe
78	GBR	United Kingdom	Western Europe

79	CHE	Switzerland	Western Europe
80	NOR	Norway	Western Europe
81	XEF	Rest of European Free Trade Association	Western Europe
82	ALB	Albania	Western Europe
83	BGR	Bulgaria	Economies in Transition (Eastern Europe and former Soviet Union)
84	BLR	Belarus	Economies in Transition (Eastern Europe and former Soviet Union)
85	HRV	Croatia	Economies in Transition (Eastern Europe and former Soviet Union)
86	ROU	Romania	Economies in Transition (Eastern Europe and former Soviet Union)
87	RUS	Russian Federation	Economies in Transition (Eastern Europe and former Soviet Union)
88	UKR	Ukraine	Economies in Transition (Eastern Europe and former Soviet Union)
89	XEE	Rest of Eastern Europe	Economies in Transition (Eastern Europe and former Soviet Union)
90	XER	Rest of Europe	Economies in Transition (Eastern Europe and former Soviet Union)
91	KAZ	Kazakhstan	Economies in Transition (Eastern Europe and former Soviet Union)
92	KGZ	Kyrgyztan	Economies in Transition (Eastern Europe and former Soviet Union)
93	XSU	Rest of Former Soviet Union	Economies in Transition (Eastern Europe and former Soviet Union)
94	ARM	Armenia	Economies in Transition (Eastern Europe and former Soviet Union)
95	AZE	Azerbaijan	Economies in Transition (Eastern Europe and former Soviet Union)
96	GEO	Georgia	Economies in Transition (Eastern Europe and former Soviet Union)
97	BHR	Bahrain	Middle East and North Africa
98	IRN	Iran, Islamic Republic of	Middle East and North Africa
99	ISR	Israel	Middle East and North Africa
100	JOR	Jordan	Middle East and North Africa
101	KWT	Kuwait	Middle East and North Africa
102	OMN	Oman	Middle East and North Africa
103	QAT	Qatar	Middle East and North Africa
104	SAU	Saudi Arabia	Middle East and North Africa
105	TUR	Turkey	Middle East and North Africa
106	ARE	United Arab Emirates	Middle East and North Africa
107	XWS	Rest of Western Asia	Middle East and North Africa
108	EGY	Egypt	Middle East and North Africa
109	MAR	Morocco	Middle East and North Africa
110	TUN	Tunisia	Middle East and North Africa
111	XNF	Rest of North Africa	Middle East and North Africa
112	BEN	Benin	Middle East and North Africa
113	BFA	Burkina Faso	Middle East and North Africa

114	CMR	Cameroon	sub-Saharan Africa
115	CIV	Côte d'Ivoire	sub-Saharan Africa
116	GHA	Ghana	sub-Saharan Africa
117	GIN	Guinea	sub-Saharan Africa
118	NGA	Nigeria	sub-Saharan Africa
119	SEN	Senegal	sub-Saharan Africa
120	TGO	Togo	sub-Saharan Africa
121	XWF	Rest of Western Africa	sub-Saharan Africa
122	XCF	Rest of Central Africa	sub-Saharan Africa
123	XAC	South Central Africa	sub-Saharan Africa
124	ETH	Ethiopia	sub-Saharan Africa
125	KEN	Kenya	sub-Saharan Africa
126	MDG	Madagascar	sub-Saharan Africa
127	MWI	Malawi	sub-Saharan Africa
128	MUS	Mauritius	sub-Saharan Africa
129	MOZ	Mozambique	sub-Saharan Africa
130	RWA	Rwanda	sub-Saharan Africa
131	TZA	Tanzania, United Republic of	sub-Saharan Africa
132	UGA	Uganda	sub-Saharan Africa
133	ZMB	Zambia	sub-Saharan Africa
134	ZWE	Zimbabwe	sub-Saharan Africa
135	XEC	Rest of Eastern Africa	sub-Saharan Africa
136	BWA	Botswana	sub-Saharan Africa
137	NAM	Namibia	sub-Saharan Africa
138	ZAF	South Africa	sub-Saharan Africa
139	XSC	Rest of South African Customs Union	sub-Saharan Africa
140	XTW	Rest of the World	sub-Saharan Africa

Supplementary Table 2 Details on model configuration

**WRF v3.9**	
Horizontal resolution	27km
Number of sigma level	26
Longwave Radiation	Rapid Radioactive Transfer Model (RRTM)
Shortwave Radiation	Dudhia scheme
Microphysics	WRF Single-Moment 6-class (WSM6)
Land-surface	Noah
Advection	global mass-conserving scheme
Planetary boundary layer (PBL) scheme	MRF
Cumulus option	Kain-Fritsch
**CMAQv5.0.2**	
Horizontal resolution	27km
Number of sigma level	18
Gas-phase chemistry	Carbon Bond 05 (CB05)
Aerosol module	AERO6
Horizontal advection module	Yamo
Vertical diffusion module	Asymmetric Convective Model version 2 (ACM2)
Photolysis calculation module	In-line
CMAQ cloud module	ACM
C CTM generalized -coordinate driver modul	Yamartino
Vertical layer Number	18 layers

Supplementary Table 3 Statistics of model performances

	1 hour average O_3		Highest daily maximum 8 hour average O_3	
**Indicator**	**Jul 2013**	**Oct 2013**	**Jul 2013**	**Oct 2013**
NMB(%)	15.11	9.70	-1.26	-14.42
NME(%)	54.02	55.26	26.22	26.47
R	0.55	0.57	0.70	0.68

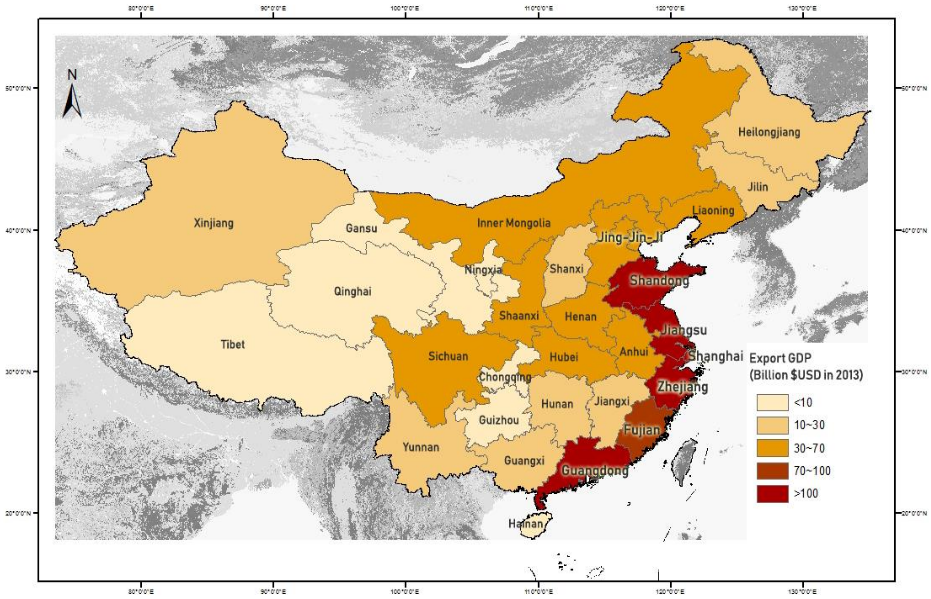

Supplementary Figure 1 Gross Domestic Product of export by provinces in 2013

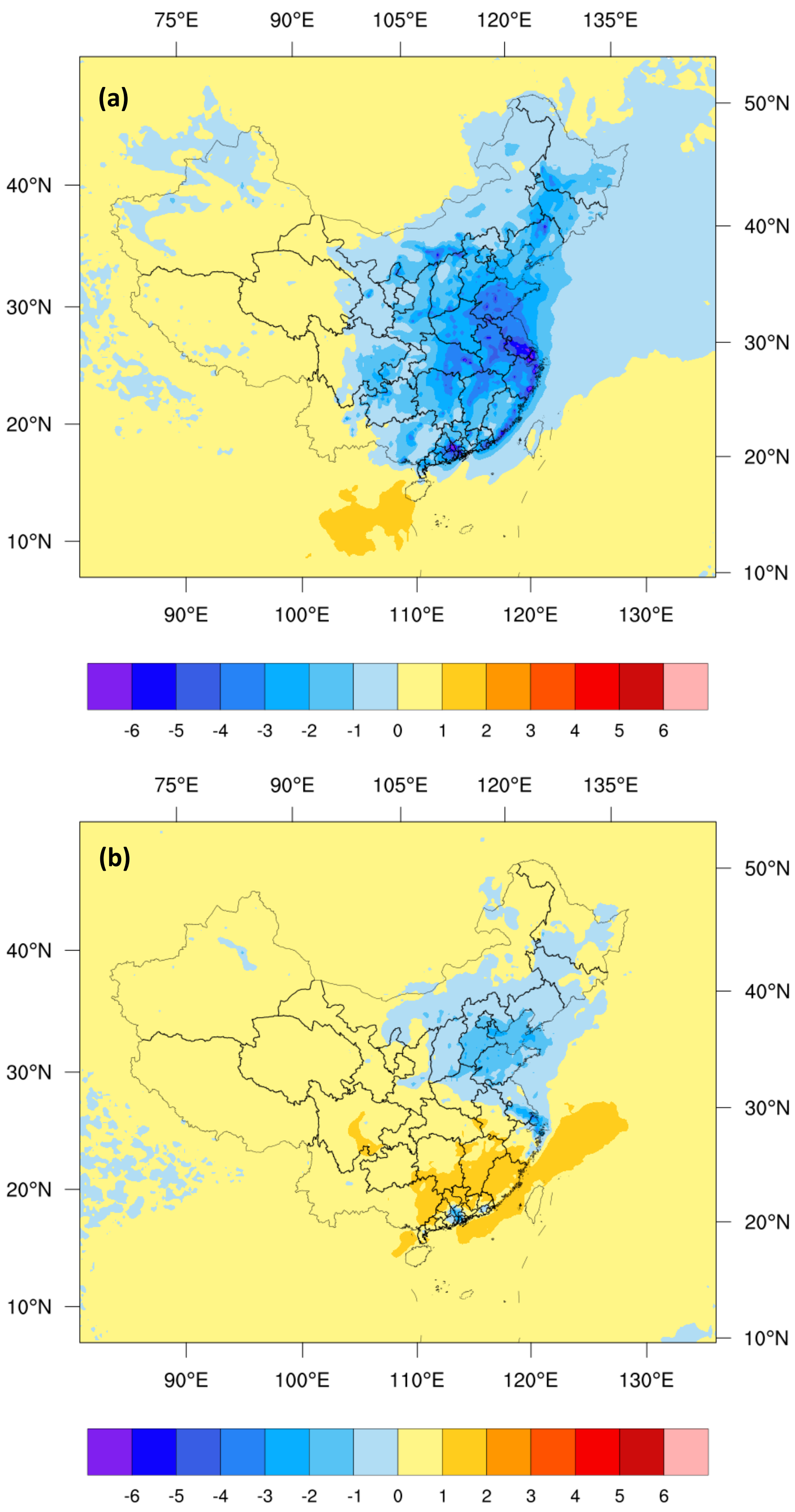

Supplementary Figure 2 Export concentration to MAX 8h O_3 in (a) January and (b) April, 2013 (ug m^{-3})

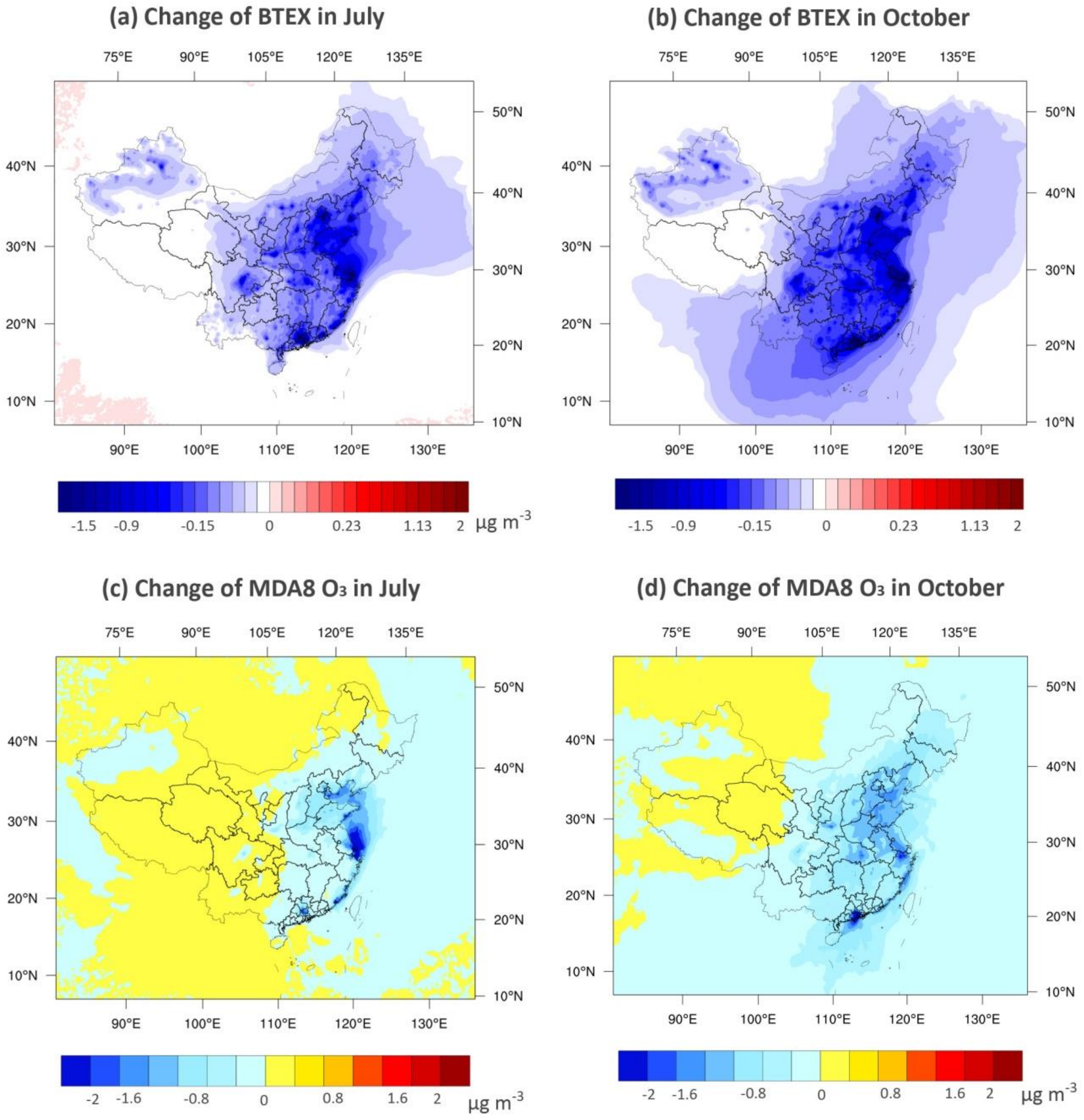

Supplementary Figure 3 Change of BTEX and MAX 8h O_3 by cleaner production practices only in export capacities

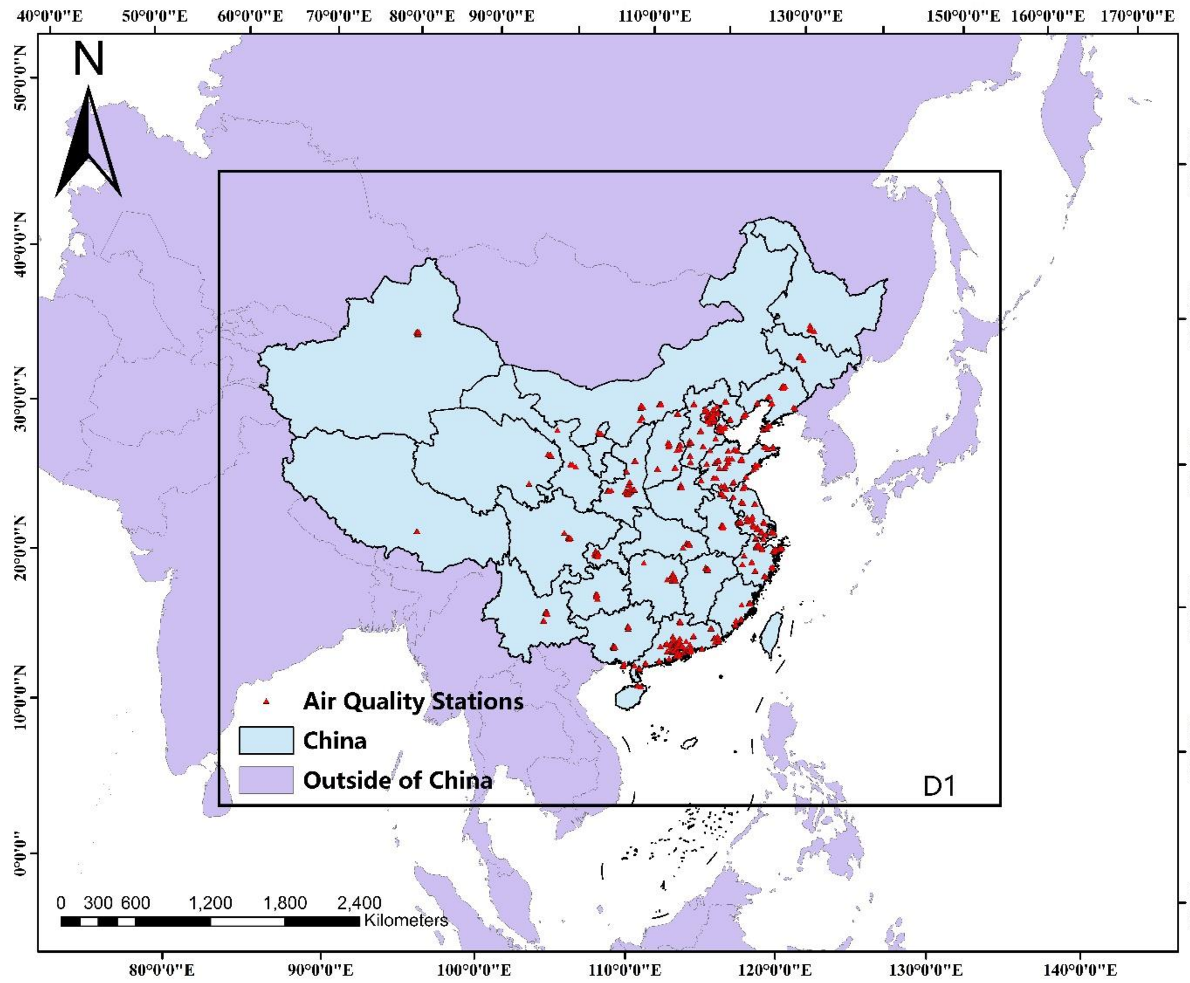

Supplementary Figure 4 Locations of ambient ozone monitoring sites in China

Supplementary References

[1] D. Thaxton, C.; Sherman, J. P.; Krintz, I. A.; Scher, A.; Ross, D.; Schlesselman, "Optimizing Weather Research and Forecasting model parameterizations for boundary-layer turbulence production and dissipation over the Southern Appalachians," in *American Geophysical Union, Fall Meeting 2017*, 2017.

[2] The institute for the Environment, the University of North Carolina at Chapel Hill. SMOKE v4.6 User's Manual. Available via https://www.cmascenter.org/smoke/documentation/4.6/manual_smokev46.pdf. (accessed on 27 September, 2018).

[3] C. Hong, Q. Zhang, Y. Zhang, Y. Tang, D. Tong, and K. He, "Multi-year downscaling application of two-way coupled WRF v3 . 4 and CMAQ v5 . 0 . 2 over east Asia for regional climate and air quality modeling : model evaluation and aerosol direct effects," *Geosci. Model Dev.*, vol. 10, pp. 2447–2470, 2017.

[4] C. Emery *et al.*, "Recommendations on statistics and benchmarks to assess photochemical model performance," *J. Air Waste Manage. Assoc.*, vol. 67, no. 5, pp. 582–598, 2017.

[5] J. Hu, J. Chen, Q. Ying, and H. Zhang, "One-year simulation of ozone and particulate matter in China using WRF / CMAQ modeling system," *Atmos. Chem. Phys.*, vol. 16, pp. 10333–10350, 2016.

[6] Y. Zhang, X. Zhang, L. Wang, Q. Zhang, and F. Duan, "Application of WRF / Chem over East Asia : Part I . Model evaluation and intercomparison with MM5 / CMAQ," *Atmos. Environ.*, vol. 124, pp. 285–300, 2016.

[7] X. Liu *et al.*, "Understanding of regional air pollution over China using CMAQ , part I performance evaluation and seasonal variation," *Atmos. Environ.*, vol. 44, pp. 2415–2426, 2010.
